# P230: The effect of health care-associated infections in the outcome of patients with heart failure

**DOI:** 10.1186/2047-2994-2-S1-P230

**Published:** 2013-06-20

**Authors:** A Spyrou, J Trikilis, C Panagiotou

**Affiliations:** 1 Coronary Care Unit, Onassis Cardiac Surgery Center, Athens, Greece

## Introduction

The patients hospitalized due to heart failure have higher percentage of contacting diseases and mortality rate when compared to other groups of patients, a fact that could be partially attributed to co-existing with health care-associated infections (HCAI).

## Objectives

The purpose of the current study is the investigation of both the impact of HCAI on patients with heart failure and the correlation of the infection and the outcome of the disease.

## Methods

70 patients with heart failure who were hospitalized in the Cardiological ICU of a Cardiological/Cardiosurgical Centre from January 2011 until December 2011 out of a total of 95 patients; the other 25 did not meet the specified criteria for their inclusion in the study. The HCAI were defined on the basis of the criteria of the US Centre for Disease Control (CDC). The statistics software Statistical Package for Social Sciences (SPSS) v 17 was utilized for the data analysis.

## Results

8.57% of the patients with heart failure presented an HSPA related infection. The patients who contacted the infection had a lengthier stay in the Cardiological ICU (p=0.04) and a lengthier overall hospitalization period (p=0.04) when compared to the patients without infection. The mortality rate of the patients with infection was comparatively higher to that of the patients without infection (53% vs. 12%, p<0.001).

## Conclusion

The infections have a negative impact on the percentage of contacting diseases and mortality rate of patients hospitalized in the Cardiological ICU with heart failure. Further investigation of the correlation of HCAI and heart failure, as the development of knowledge in this specific sector can offer significant results in the therapeutic approach of these patients.

## Disclosure of interest

None declared

